# Pectin nanoparticles loaded with nitric oxide donor drug: A potential approach for tissue regeneration

**DOI:** 10.1016/j.ijpx.2024.100244

**Published:** 2024-04-02

**Authors:** Noha I. Elsherif, Abdulaziz M. Al-Mahallawi, Iman Saad Ahmed, Rehab N. Shamma

**Affiliations:** a Department of Pharmaceutics and Pharmaceutical Technology, Faculty of Pharmacy, Heliopolis University, Cairo 11785, Egypt; b Department of Pharmaceutics and Industrial Pharmacy, Faculty of Pharmacy, Cairo University, Cairo 12613, Egypt; c School of Life and Medical Sciences, University of Hertfordshire Hosted by Global Academic Foundation, New Administrative Capital, Cairo 11835, Egypt; d Department of Pharmaceutics & Pharmaceutical Technology, College of Pharmacy, University of Sharjah, Sharjah 27272, United Arab Emirates; e Research Institute for Medical and Health Sciences, University of Sharjah, Sharjah 27272, United Arab Emirates

**Keywords:** Pectin, Nebivolol hydrochloride, Wound healing, Tissue regeneration, Nanoparticle

## Abstract

The process of wound healing and tissue regeneration involves several key mechanisms to ensure the production of new tissues with similar cellular functions. This study investigates the impact of pectin, a natural polysaccharide, and nebivolol hydrochloride (NBV), a nitric oxide (NO) donor drug, on wound healing. Utilizing ionotropic gelation, NBV-loaded pectin nanoparticles were developed following a 2^2^3^1^ full factorial design. The optimized formulation, determined using Design expert® software, exhibited an encapsulation efficiency percentage of 70.68%, zeta potential of −51.4 mV, and a particle size of 572 nm, characterized by a spherical, discrete morphology. An *in vivo* study was conducted to evaluate the effectiveness of the optimal formulation in wound healing compared to various controls. The results demonstrated the enhanced ability of the optimal formulation to accelerate wound healing. Moreover, histopathological examination further confirmed the formulation's benefits in tissue proliferation and collagen deposition at the wound site 15 days post-injury. This suggests that the developed formulation not only promotes faster healing but does so with minimal side effects, positioning it as a promising agent for effective wound healing and tissue regeneration.

## Highlights

• Recognizing that wound healing is a complex interplay of cellular events, this study aimed to harness the benefits of both pectin and nitric oxide donor nebivolol hydrochloride.
• Ionotropic gelation was used to fabricate nebivolol-loaded pectin nanoparticles, by crosslinking pectin using sodium tripolyphosphate.
• The influence of different processing variables on the properties of nebivolol-loaded pectin nanoparticles were investigated, as well as examining the morphology of the optimized pectin nanoparticles using TEM.
• The optimized nebivolol-loaded pectin nanoparticles were compared against both positive and negative controls to evaluate their effects on tissue healing and regeneration.

## Graphical abstract

**Figure:**
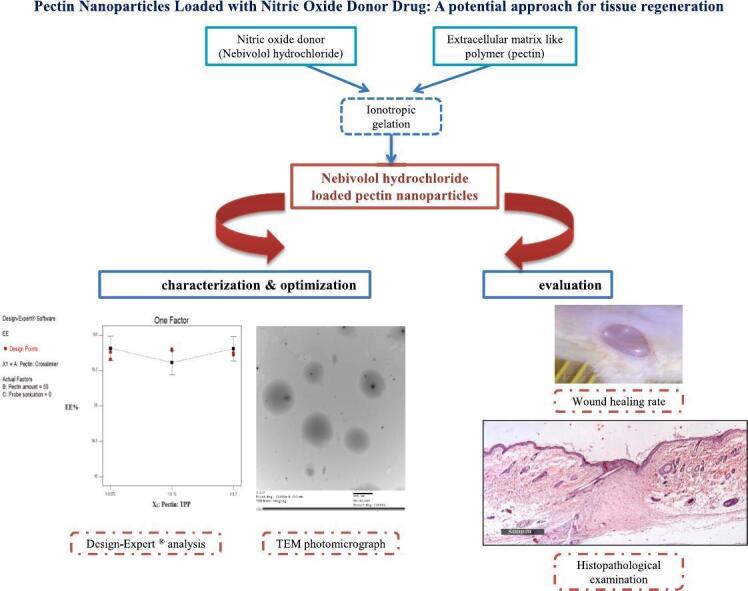

## 1 Introduction

Drug repurposing, a strategy focused on exploring new therapeutic uses for already established drugs, has been adopted by several pharmaceutical companies (Mittal and Mittal, 2021). This strategy has proven to be highly efficient and cost-effective, saving time and significantly reducing the risk of failure (Mittal and Mittal, 2021), making drug repurposing a reasonable and realistic opportunity. Nebivolol hydrochloride (NBV) is a lipophilic, third-generation beta-blocker and nitric oxide (NO) donor, officially approved for the management of hypertension. NBV exerts its vasodilating action by releasing cardiovascular endothelial NO (Jatav et al., 2013), along with its conventional beta-blocking effects (Hilas and Ezzo, 2009). Owing to its classification as a Class II drug in the Biopharmaceutics Classification System (BCS), numerous scientists have attempted to improve its solubility through various modified drug delivery systems (Thadkala et al., 2015; Al-Dhubiab et al., 2019). To improve the solubility and bioavailability of NBV, it has been formulated into various nanoscale drug delivery systems, including solid lipid nanoparticles (Üstündağ-Okur et al., 2016), nanocrystals (Al-Dhubiab et al., 2019), chitosan nanoparticles (Sharma et al., 2018), and lipospheres (Hanif et al., 2019). Additionally, diverse preparation techniques have been employed to optimize these formulations (Pravala et al., 2013; Shah et al., 2014). Recent studies have revealed that nitric oxide (NO), a small endogenously produced diffusible molecule (Ahmed et al., 2022), exhibits increased levels in wound sites (Schaffer et al., 1996; Schäffer et al., 1997; Luo and Chen, 2005). NO plays a crucial role in wound healing processes as it inhibits platelet aggregation, deactivates monocytes, and stimulates the proliferation of endothelial cells (Ahmed et al., 2022). These actions highlight its significant contribution to accelerating wound closure and promoting healing (Frank et al., 2002). Several studies have highlighted the role of exogenous NO in wound healing, emphasizing its small size which facilitates easier penetration, vasodilation and promotion of angiogenesis (Tavares et al., 2022). Recognizing its efficacy, one of the initial approaches involved applying gaseous NO directly to skin wounds (Shekhter et al., 2005; Pinto et al., 2022). Further research has investigated NO's wound healing properties using various carriers, such as probiotic patches (Jones et al., 2010), hybrid hydrogel-glass nanoparticles (Friedman et al., 2008) and amine-functionalized silica nanoparticles (Shin and Schoenfisch, 2008). In this context, NBV becomes particularly relevant. Previous studies have demonstrated NBV's effectiveness in slowing diabetic neuropathy, restoring endothelial function in diabetic wounds (Pandit et al., 2017), and enhancing wound healing rates (Ulger et al., 2016). These benefits can be achieved either by using NBV in a standalone formulation (Ulger et al., 2016), or by incorporating it into modified drug delivery systems (Pandit et al., 2017; Elsherif et al., 2021).

Natural polysaccharides are gaining increasing attention in the development of drug delivery systems due to their ease of production and reproducibility. Their inherent capacity to undergo crosslinking facilitates the creation of nanoparticulate systems and hydrogels, making them particularly attractive for pharmaceutical applications (Alvarez-Lorenzo et al., 2013). Their biocompatibility, bioabsorbability, and biodegradability, coupled with a lack of immune-stimulatory activities, render natural polysaccharides ideal candidates for innovative drug delivery systems. (Patrulea et al., 2015). Pectin is a hydrophilic anionic carbohydrate polymer, naturally present as a primary structural component in plant cell walls (Mishra et al., 2012). It is a non-starch polysaccharide that is primarily extracted from citrus and apple peels, using a process involving low pH and high temperature, yielding mainly α-galacturonan (Voragen et al., 2009). Chemically, it is composed of poly α 1–4- galacturonic acids, with varying degree of methylation in its carboxylic acid residues (Grant et al., 1973). Pectin is freely soluble in water (Jonassen et al., 2013; Cao et al., 2020), and has been extensively used in the food industry, notably as a gelling agent in jams and jellies (Mishra et al., 2012), and as a thickening agent and colloidal stabilizer in acidified milk drinks and yoghurt (Burapapadh et al., 2016). Its widespread use is also due to its ability to be degraded by microbial enzymes present in the colon (Mishra et al., 2012), confirming its biocompatibility and biodegradability (Wakerley et al., 1996; Jonassen et al., 2013; Bostancı et al., 2022).

Pectin's diverse advantages have led to its extensive recent use in various applications. It has been utilized to coat and encapsulate metal nanoparticles, preventing their aggregation due to its high anionic charge (Nemiwal et al., 2021). Additionally, pectin serves as a reducing agent in the green synthesis of metallic nanoparticles (Devasvaran and Lim, 2021). In the field of nanotechnology, pectin has been employed in creating nanofibers through electrospinning (Mamidi et al., 2022a, Mamidi et al., 2022b), often combined with synthetic polymers, resulting in nanofibers suitable for biological applications. Pectin has also been integrated with carbonaceous nanomaterials (Mamidi et al., 2022a, Mamidi et al., 2022b) like carbon nanotubes and graphene forming biodegradable film composites renowned for their strength and water resistance (Farahnaky et al., 2018).

In the context of wound healing, pectin's ability to absorb wound exudate (Bostancı et al., 2022), makes it particularly useful. It has been applied in various forms, including hydrogels (Giusto et al., 2017), patches (Andriotis et al., 2020), films (Zulema et al., 2020) and nanoparticles (Rajapaksha et al., 2020), either alone or in combination with other polymers and bioactive agents (Birch and Schiffman, 2014; Alipour et al., 2019; Oveissi et al., 2020). This escalating interest in pectin is also attributed to its bioactive properties; it promotes the proliferation of B cells and the secretion of interleukin-1β by macrophages, which are crucial processes in wound healing (Rajapaksha et al., 2020).

Recognizing that wound healing is a complex interplay of cellular events, this study aimed to harness the benefits of both pectin and NBV. We developed NBV-loaded pectin nanoparticles to evaluate their potential for wound healing enhancement. Ionotropic gelation was used to fabricate NBV-loaded pectin nanoparticles, by crosslinking pectin using sodium tripolyphosphate. The factorial design used in this process was evaluated and optimized using Design® Expert software to assess the different selected factors. The morphology of the optimized nanoparticles was examined using transmission electron microscopy, and its solid state was characterized through differential scanning calorimetry and X-ray diffraction. To assess the effectiveness of the optimized formulation, we conducted an *in vivo* excisional wound study on rats. Here, the optimized NBV-loaded pectin nanoparticles were compared against both positive and negative controls to evaluate their individual and combined effects on tissue healing and regeneration. Furthermore, a histopathological study was conducted on isolated skin samples to assess the optimal formulation's impact on cellular proliferation and collagen reconstruction.

While pectin nanoparticles and the use of NBV for tissue regeneration are well-established in the field, our developed formulation stands out for its innovative approach. Unlike conventional methods that require incorporating nanoparticles into a cream or ointment formulation for topical application, our system uniquely exploits the gel-like nature of the developed drug-loaded pectin nanoparticles. This innovative strategy not only simplifies application but also enhances drug delivery efficiency and potentially increases the drug's effectiveness at the wound site, while reducing costs.

## 2 Materials and methods

### 2.1 Materials

Nebivolol hydrochloride (NBV) was generously provided by Marcyrl Pharmaceutical Industries (Cairo, Egypt). Pectin pure powder (molecular weight 300,000-1000,000; degree of esterification 63–66%) was purchased from Alpha Chemika (Andheri west, Mumbai, India). Tri-polyphosphate sodium (TPP) was purchased from Sigma–Aldrich Co. (St. Louis, MO, USA). Methyl alcohol, potassium dihydrogen phosphate (KH_2_PO_4_), disodium hydrogen phosphate (Na_2_HPO_4_), sodium chloride (NaCl), Tween 80, and potassium chloride (KCl) were purchased from Adwic, El-Nasr Pharmaceutical Co. (Abou-Zaabal, Egypt). Sodium chloride infusion BP 2014 (Sodium chloride 0.9% *w/v*, Normal saline) was supplied from a local pharmacy and stored according to the package information leaflet.

### 2.2 Preparation of NBV-loaded pectin nanoparticles

NBV-loaded pectin nanoparticles (NBV-loaded-pectin-NP) were prepared using the ionotropic gelation method (Calvo et al., 1997; Sriamornsak and Nunthanid, 1999; Chinnaiyan et al., 2018; Dogan Ergin et al., 2021). The required amount of pectin powder was accurately weighed using an electrical balance (ViBRA HT, Antielectrostatic, Japan), and then dissolved in distilled water to prepare various concentrations of pectin solutions. The prepared solutions were then filtered using a syringe filter 0.22 μm nylon (Millipore, USA) for further use (Pistone et al., 2017; Chinnaiyan et al., 2018). For the preparation, the volume corresponding to the required amount of pectin (10 mL of 1% *w/v* pectin in distilled water for 100 mg pectin, and 10 mL of 0.5% *w/v* pectin in distilled water for 50 mg pectin) was measured and placed in a beaker on a magnetic stirrer (WiseStir, Wisd Lab. Instruments, Tulsa, OK, USA). NBV was then accurately weighed, dissolved in 4 mL methanol, and added to the respective pectin solution on the magnetic stirrer, set at 1000 rpm at room temperature for 1 min. The appropriate amount of TPP was dissolved in 2 mL of distilled water, and then gradually added to the beaker in a drop wise manner. The resulting dispersion was continuously stirred at 1000 rpm for 30 min at 50 °C (Dogan Ergin et al., 2021), ensuring the complete evaporation of the methyl alcohol (resulting in a total volume of approximately 12 mL). The resulting NBV-loaded pectin nanoparticles were then stored overnight in a refrigerator at 5 °C for subsequent use.

Certain samples underwent probe sonication for 3 min to assess its impact on reducing particle size (PS). For this process, after sample preparation, each sample was transferred into a glass beaker, which was then set in an ice bath. Subsequently, a probe sonicator (Ultrasonic processor VCX750, Newtown, USA) was employed for the sonication. Post-sonication, these samples were stored overnight in the refrigerator at 5 °C before being used.

### 2.3 Characterization of NBV-loaded pectin nanoparticles

#### 2.3.1 Determination of NBV encapsulation efficiency (EE %)

The NBV-loaded pectin nanoparticles (NBV-loaded-pectin-NP) were analyzed for their encapsulated NBV content using the indirect method (Sharma et al., 2012; Chinnaiyan et al., 2018). For this analysis, 1 mL samples of the prepared NBV-loaded-pectin-NP were subjected to centrifugation in a cooling centrifuge (Sigma 3–30 KS, Sigma Laborzentrifugen GmbH, Germany) set at 4 °C and 15,000 rpm for 1 h. The supernatant was collected post-centrifugation and the remaining precipitate was washed with distilled water and re-centrifuged under the same conditions for 15 min. This supernatant, together with the supernatant obtained from the initial centrifugation were combined, diluted to a total volume of 10 mL with methanol, and then measured spectrophotometrically at the predetermined λ_max_ of 281.7 nm (Shimadzu, model UV-1601 PC, Kyoto, Japan) based on a previously established standard curve (Meyyanathan et al., 2010; Rao et al., 2010; Elsherif et al., 2021). The EE % was calculated using the following equation (all measurements were performed in triplicate for each sample at 25 °C):

$$\mathrm{EE}\%=\frac{\left(\mathrm{Theoretical amount of}\;\mathrm{NBH}-\mathrm{Measured amount of}\;\mathrm{NBH}\right)}{\mathrm{Theoretical amount of}\;\mathrm{NBH}}\;x\;100$$

#### 2.3.2 Determination of particle size distribution

The particle size (PS) and polydispersity index (PDI) of the prepared NBV-loaded-pectin-NP were determined using the light scattering technique with a Zetasizer Nano ZS (Malvern Instruments, Malvern, UK). Prior to each measurement, each formulation was appropriately diluted with distilled water at a ratio of 1:10 *v/v*), following the methodology outlined in previous studies (Sharma et al., 2012; Dogan Ergin et al., 2021; El Said et al., 2022). All measurements were performed in triplicate for each sample at 25 °C (Abdelbary et al., 2019; Albash et al., 2021a, Albash et al., 2021b; Albash et al., 2021a, Albash et al., 2021b).

#### 2.3.3 Determination of zeta potential

The zeta potential (ZP) of the different NBV-loaded-pectin-NP (without centrifugation) was assessed to determine the overall charges acquired by the nanoparticles, an important indicator of their stability (Abdelbary and AbouGhaly, 2015). For this measurement, 0.1 mL of each formulation was diluted to 10 mL with distilled water and analyzed using a Zetasizer Nano-ZS. Each measurement was performed in triplicate at 25 °C (Tayel et al., 2015; Ahmed et al., 2019; Al-mahallawi et al., 2021b). It was assumed for these measurements that the viscosity of the samples was equivalent to that of water, as suggested by previous studies (Wang et al., 2007; Al-Mahallawi et al., 2021a).

### 2.4 Experimental design

The design of choice for preparing the NBV-loaded-pectin-NP was a 2^2^ 3^1^ full factorial design, utilizing Design Expert® software (Version 10, Stat-Ease Inc. Minneapolis, MN, USA). Subsequently, an analysis of variance (ANOVA) test was applied to assess the significance of each factor (Sharma et al., 2012). The key independent variables for optimization included the pectin to TPP ratio (X_1_), the amount of pectin (X_2_), and the time of probe sonication (X_3_). These factors were chosen to optimize the dependent variables or responses, namely, Encapsulation Efficiency Percent (EE %, Y_1_), Particle Size (PS, Y_2_), and Zeta Potential (ZP, Y_3_), as detailed in Table 1. The initial design comprised 12 experimental runs and statistical significance was determined at *p* ≤ 0.05. The results of the experimental runs and the corresponding measured responses are shown in Table 2.

**Table 1:** The independent variables (factors) and dependent variables (responses) for the optimization of NBV-loaded pectin nanoparticles using a 2^2^3^1^ full factorial design.

Factors (independent variables)	Levels		
X_1_: The pectin to TPP ratio	1:0.075	1:0.15	1:0.3
X_2_: The amount of pectin (mg)	50		100
X_3_: The time of probe sonication (min)	0		3


Responses (dependent variables)	Constraints
Y_1_: Entrapment efficiency (%)	Maximum
Y_2_: Particle size (nm)	Minimum
Y_3_: Zeta potential (mV)	Maximum

Abbreviations: TPP: sodium tripolyphosphate, NBV: nebivolol hydrochloride.

**Table 2:** The experimental design and measured responses for the optimization of NBV-loaded-pectin nanoparticles.

Formula	Independent Variables			Dependent Variables			
	X_1_: Pectin to TPP ratio *(w/w)*	X_2_: Amount of pectin (mg)	X_3_: Time of probe sonication (min)	Y_1_: EE % ⁎	Y_2_: PS (μm) ⁎	Y_3:_ ZP (mV)⁎	PDI ⁎
T1	1:0.075	50	0	88.30 ± 2.96	2.096 ± 0.084	−37.7 ± 1.83	0.52 ± 0.05
T2	1:0.15	50	0	91.87 ± 0.64	3.773 ± 0.375	−31.6 ± 0.14	0.42 ± 0.02
T3	1:0.3	50	0	89.36 ± 1.04	1.854 ± 0.159	−51.1 ± 0.91	0.60 ± 0.05
T4	1:0.075	100	0	87.54 ± 2.02	3.220 ± 0.082	−43.7 ± 0.56	0.70 ± 0.01
T5	1:0.15	100	0	55.10 ± 1.55	1.619 ± 0.072	−41.0 ± 1.62	0.41 ± 0.04
T6	1:0.3	100	0	84.62 ± 2.18	4.981 ± 0.101	−51.5 ± 0.21	0.66 ± 0.06
T7	1:0.075	50	3	56.52 ± 2.58	0.694 ± 0.037	−37.5 ± 1.55	0.39 ± 0.02
T8	1:0.15	50	3	16.01 ± 0.41	0.699 ± 0.040	−31.4 ± 0.07	0.39 ± 0.11
T9	1:0.3	50	3	68.26 ± 2.77	0.493 ± 0.027	−50.8 ± 0.49	0.30 ± 0.03
T10	1:0.075	100	3	57.96 ± 5.37	0.942 ± 0.031	−43.8 ± 0.35	0.46 ± 0.05
T11	1:0.15	100	3	30.03 ± 0.25	0.559 ± 0.002	−41.2 ± 1.34	0.35 ± 0.01
T12	1:0.3	100	3	70.68 ± 3.79	0.572 ± 0.021	−51.4 ± 0.07	0.21 ± 0.02

Abbreviations: TPP: sodium tripolyphosphate, EE %: entrapment efficiency percentage, PS: particle size, ZP: zeta potential, PDI: polydispersity index.

⁎ Data are represented as mean (*n* = 3) ± S.D.

### 2.5 Characterization of the optimized NBV-loaded pectin nanoparticles

#### 2.5.1 *In-vitro* release

Using a thermostatically controlled water bath shaker (Gesellschaft Laboratories, Berlin, Germany), the dialysis bag diffusion technique was employed to assess the release of NBV from the optimized NBV-loaded-pectin-NP and compared it to NBV aqueous suspension as a control (Kumbhar and Pokharkar, 2013; Chinnaiyan et al., 2018). To ensure sink conditions, the chosen release media was phosphate buffer saline (pH 7.4) containing 1% Tween 80 (Pandit et al., 2017; Elsherif et al., 2021). The cellulose dialysis membrane (Visking® dialysis tubing, diameter 21 mm, MWCO 12,000–14,000 Da, Serva, Heidelberg, Germany) was soaked overnight in the release medium, to ensure its complete hydration. After centrifugation of the sample, an amount of the precipitate equivalent to 1 mg of encapsulated NBV from the optimized NBV-loaded-pectin-NP was suspended in 1 mL of distilled water inside the dialysis bag; and then securely tied at both ends. Similarly, 1 mL of the control (NBV aqueous suspension at 0.1% *w/v*) was placed into another dialysis bag, also tied securely at both ends. The dialysis bags were immersed in beakers containing 100 mL of the chosen release media and subjected to agitation using a thermostatically controlled shaker (Memmert, Buchenbach, Germany) operating at 100 rpm and maintained at 32 ± 0.5 °C. At predetermined time intervals (0.5 h, 1 h, 2 h, 3 h, 4 h, 5 h, 6 h, 7 h, 8 h, and 24 h), samples of 3 mL from the release media were collected and quantitatively assessed spectrophotometrically at the predetermined λ_max_ of 283.0 nm. To maintain a constant volume of the release media, each withdrawn sample was immediately replaced by an equal volume of clear release medium. Each experiment was performed in triplicate.

The Korsmeyer−Peppas model was used to analyze the release behavior of NBV from the optimized formulation. The model is described as follows (Rashidipour et al., 2019):

$$M^{t}/M^{\infty }={\mathrm{Kt}}^{n}$$

M^t^ is the quantity of NBV released in time t, M^∞^ is the quantity of NBV released at infinite time, K represents the kinetic release constant, and n is the release exponent.

#### 2.5.2 Transmission electron microscopy (TEM)

The morphology of the optimized NBV-loaded-pectin-NP was determined by TEM (Jeol JEM 1230, Tokyo, Japan). This study was focused on assessing the size, sphericity and aggregation characteristics of the NP (Sharma et al., 2012). The optimized formulation was measured using the same procedures described previously in literature (Fahmy et al., 2020; Fahmy et al., 2021). In preparation for examination, a 2.5% phosphotungstic acid stain was applied followed by the deposition of one drop of the sample onto a carbon-coated copper grid. After air drying at room temperature for 10 min, the samples were examined under TEM at magnifications of 10,000×, 15,000× and 30,000×.

#### 2.5.3 Differential Scanning Calorimetry (DSC)

DSC (Mettler-Toledo International Inc., Columbus, OH, USA) was employed to assess the thermal properties of the dry selected formulation; in comparison with its corresponding physical mixture, pure NBV, and pectin as standard references for comparison (Pramanik and Ganguly, 2018). Lyophilization of the optimized formulation was conducted prior to its DSC evaluation, using a freeze dryer (Novalyphe-NL 500, Halprook, NY, UA). The selected formulation was freezed at −20 °C, followed by sublimation for 48 h in the freeze dryer. Approximately 5 mg of the dried samples were weighed and analyzed in hermetically sealed aluminum pans which were heated at a scanning rate of 10 °C/min between 25 °C–415 °C, with nitrogen serving as the blanket gas during the analysis.

#### 2.5.4 X-Ray Diffractometry (XRD)

The XRD was conducted to determine the crystallinity of the encapsulated NBV inside the optimized lyophilized NBV-loaded-pectin-NP. The lyophilized sample, along with its corresponding physical mixture, pure NBV, and pectin were evaluated using X-ray diffractometer (XGEN-4000, Scintag Corp., Sunnyvale, CA, USA). Cu Kα radiation at 1.542 Å, with a voltage set at 40 kV and a current at 20 mA, was employed for the XRD analysis. The instrument was configured for continuous scanning across a 2θ range from 5° to 50°, at a scanning rate of 6°/min (Piao et al., 2011).

### 2.6 *In vivo* animal study

#### 2.6.1 Sterilization using gamma radiation

Prior to conducting the *in vivo* animal studies, the samples used were sterilized to ensure the sterility of the samples before application to the animals (Morsi et al., 2019). The samples used were the optimized nanoparticles obtained through the cooling centrifugation separation process. The method of choice for sterilization was the gamma radiation method at a dose of 10 kGy (Maged et al., 2019; Morsi et al., 2019), and sterilization was conducted at the Egyptian Atomic Energy Authority. The sterilized samples were NBV aqueous suspension (B), blank optimized pectin nanoparticles (C) and the optimized NBV-loaded-pectin-NP (D).

NBV aqueous suspension (0.1% *w/v*) was prepared by suspending a specific amount of NBV in 10 mL of distilled water, corresponding to the optimized NBV-loaded formulation. Conversely, the blank optimized pectin nanoparticles were formulated using the same methodology outlined in section 2.2 without the addition of NBV.

#### 2.6.2 Animal model

The animal study aimed to evaluate the individual and combined effects of NBV and pectin on wound healing, comparing them to a no-treatment control group. This study was conducted according to the protocol approved by the Research Ethics Committee of the Faculty of Pharmacy, Cairo University, Cairo, Egypt (REC, PI 1965, 27 April 2017). The study animals were divided randomly into four groups, each comprising four animals. This resulted in a total of 16 male and female Albino Sprague-Dawley rats, with weights ranging from 150 to 200 g. Prior to the study, all four animals within each group were housed together in a polycarbonate cage with free access to food (standard diet) and water (Pandit et al., 2017; Maged et al., 2019) at the Heliopolis University's animal house. The environmental conditions were controlled with a constant temperature of 25 ± 1 °C, humidity of 45–55%, and artificial illumination provided by fluorescent lights set on a 12/12 reversed light cycle. Throughout the study, the rats were checked daily for any abnormalities to ensure their health.

#### 2.6.3 Wound induction protocol

The design selected to evaluate the wound closure efficacy and tissue regeneration was the excision wound model (Nagar et al., 2016; Maged et al., 2019). To induce the wound, the animals were first anesthetized using thiopental sodium (25 mg/kg) (Sirohi and Sagar, 2019), and then using a clean razor, their back hair was meticulously shaved.

Before creating the wound, the designated wound area was defined using a permanent marker. Then, using a sterile biopsy punch needle (No. 10, Kai Industries Co., Ltd., Seki City, Japan), the wound was carefully induced on the dorsal skin, penetrating to a subcutaneous depth on the side of the spine. The shape of the wound created was a circular one, measuring 10 mm diameter. Subsequently, the rats were individually housed in separate cages to prevent further injury, leaving the wounds undressed. The rats were divided into four treatment groups, Group A served as the control group and received no treatment; Group B received the NBV aqueous suspension; Group C received the blank optimized pectin nanoparticles, whereas Group D received the optimized NVB-loaded-pectin-NP (T12).

Before applying the daily treatment, the wounds were cleaned with sterile normal saline, followed by the application of the test formulation each morning, using an amount sufficient to evenly cover the entire wound area (approximately 1 mg of NBV) (Gulcan et al., 2012; Nagar et al., 2016). The endpoint of the study was defined as the complete healing of the wound in any of the tested groups (Nagar et al., 2016; Masson-Meyers et al., 2020).

#### 2.6.4 Evaluation of wound healing progress

To monitor and assess the healing progress, the wounds were photographed using a standard mobile camera (Samsung 32 Megapixel, Japan) on days 5, 10 and 15 of the study. Additionally, routine checks for any signs of bleeding, pus, inflammation or abscess formation around the wounds were performed.

To quantify the healing progress, the remaining wound area was measured using a caliper, and calculated (Maged et al., 2019; Masson-Meyers et al., 2020) as follows:

Wound size %= $\frac{\mathrm{wound size}\;\mathrm{at}\;\mathrm{nth}\;\mathrm{day}}{\mathrm{initial wound size}}*100$

#### 2.6.5 Histopathology of wound granulating tissue

At the conclusion of the 15-day study period, we followed established procedures for rat euthanasia, using shoulder dislocation as the humane method (El-Bahy et al., 2018). Subsequently, skin samples were isolated using a sterile biopsy punch needle (No. 10), taking care to include both the dermis and hypodermis in the samples. The autopsy skin samples were then carefully trimmed using a sterile pair of scissors. They were then flushed and fixed in a 10% neutral formalin solution for a period of 72 h (Culling, 2013). The samples were then washed with distilled water followed by serial grades of ethanol washes, and finally cleared using xylene. The prepared samples were subsequently embedded in paraplast tissue embedding media. Using a rotary microtome (Leica Microsystems SM2400, Cambridge, England), the isolated sections were sliced into very thin sections, approximately 4 μm in thickness, to reveal the different skin layers within the samples. Moreover, to study the general morphological characteristics of the isolated tissue, the tissue sections were stained using Hematoxylin and Eosin (H and E) (Amrutiya et al., 2009; Culling, 2013; Pandit et al., 2017); whereas to demonstrate the dermal collagen fibers deposition, the tissue sections were stained using Masson's trichrome stain (Shrestha and Haylor, 2014; Pandit et al., 2017).The stained samples were inspected under a light microscope (Leica Microsystems GmbH, Wetzlar, Germany) (Culling, 2013).

#### 2.6.6 Statistical analysis

To establish statistically significant differences among the applied treatments concerning both wound remaining percentage and collagen fiber deposition, SPSS® version 22.0 (IBM Corporation, Chicago, Illinois, USA) was employed. The one way analysis of variance (ANOVA), followed by the least square difference (LSD) test was utilized to assess the significance of these differences. A *p*-value <0.05 was considered statistically significant.

## 3 Results and discussion

### 3.1 Preparation and optimization of NBV-loaded-pectin-NP

Previous researches have shown that various parameters and processing conditions influence the formation of pectin nanoparticles (Verma et al., 2011; Jonassen et al., 2013; Ro et al., 2015; Rashidipour et al., 2019). It was observed that the particle size of the reported formulations was in the micro range. Therefore, probe sonication was applied to the pectin nanoparticles to decrease their PS. Probe sonication time was set at 3 min to avoid subjecting the samples to excessive disruption and prevent significant loss of NBV from the nanoparticles (Andersen et al., 2013).

Sodium tripolyphosphate (TPP) has been recently used as a crosslinker with pectin to form pectin nanoparticles (Chinnaiyan et al., 2018; Rashidipour et al., 2019). Previous reports confirmed successful formation of nanoparticles between pectin and TPP (Parker et al., 1993; Auriemma et al., 2020). The mechanism of crosslinking involves the formation of strong covalent bonds between TPP ions and galacturonic acid in pectin. This occurs through ester bond formation between the hydroxyl groups of galacturonic acid and TPP (Chinnaiyan et al., 2018), leading to formation of stable pectin nanoparticles. These covalent bonds could be efficient in entrapping NBV, potentially leading to higher EE % and slower release (Jacob et al., 2020).

**Fig. 1:**
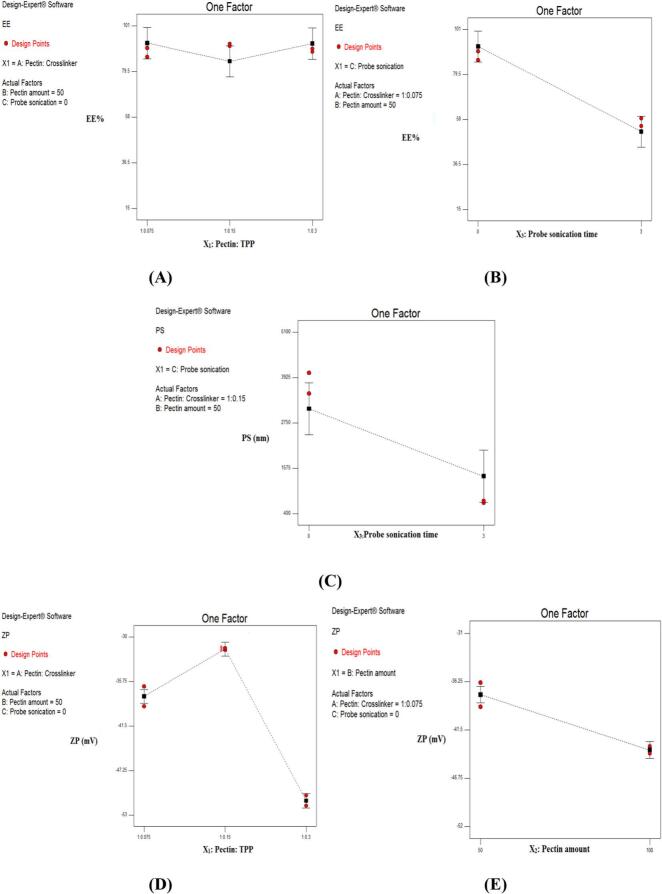
Line chart showing the effect of (A) ratio of pectin to TPP (X_1_) on EE %; (B) probe sonication time (X_3_) on EE %, (C) probe sonication time (X_3_) on PS, (D) ratio of pectin to TPP (X_1_) on ZP, and (E) amount of pectin (X_2_) on ZP of the prepared NBV-loaded pectin nanoparticles.

In this study, NBV-loaded-pectin-NP were optimized using a 2^2^3^1^ full factorial design. The configuration of the prepared 12 NBV-loaded-pectin-NP formulations which were generated using the statistical design are summarized in Table 2. The assessment of the impact of the pectin to TPP ratio (X_1_), the amount of pectin (X_2_), and the time of probe sonication (X_3_) on the produced NBV-loaded-pectin-NP resulted in EE % ranging from 16.01 to 91.87%, PS ranging from 0.49 to 4.9 μm with PDI ranging from 0.21 to 0.70 and ZP in the range of −51.5 to −31.4 mV. These broad ranges indicate that the factors selected for the optimization process had a significant impact on the response variables. The graphical representation of the relationship between the studied factors and the responses are presented in Fig. 1.

#### 3.1.1 Effect of the studied factors on entrapment efficiency (Y_1_)

The Encapsulation Efficiency (EE %) was measured indirectly due to the difficulty in dissolving the nanoparticles in methyl alcohol which resulted in a gel like precipitate. The EE % of NBV in different pectin nanoparticles ranged between 16.01 ± 0.41 and 91.87 ± 0.64% (Table 2). ANOVA results revealed significant impacts of both X_1_ and X_3_ on EE % (*p* < 0.0001 for both). Results showed that the relationship between the pectin to TPP ratio (X_1_) and EE % (Y_1_) was non-linear. Increasing the TPP amount from 1:0.075 to 1:0.15 led to a decrease in EE %, followed by an increase in EE % when the TPP amount was further increased to 1:0.3 (Fig. 1A). This suggests that the optimal level of TPP crosslinking with pectin is at an intermediate ratio (1:0.15 pectin to TPP), forming a more rigid matrix that less effectively incorporates NBV. Moreover, considering the cationic nature of NBV (Üstündağ-Okur et al., 2016) and the anionic nature of both pectin (Dogan Ergin et al., 2021) and TPP (Silvestro et al., 2020), interactions vary at different TPP levels. At lower TPP levels (1: 0.075), more pectin remains free and un-crosslinked, as indicated by the increased ZP negativity, promoting interaction with NBV and resulting in higher EE %. Conversely, at higher TPP levels (1: 0.3), excess TPP remains free and un-crosslinked, also indicated by increased ZP negativity, leading to greater interaction with cationic NBV and thus higher EE %.

Regarding the probe sonication time (X_3_), results revealed that applying 3 min sonication to the samples led to a significant decrease in EE % (Fig. 1B). This decrease in EE % is likely due to the loss of NBV from the nanoparticles during their disruption and subsequent reaggregation under ultrasonic radiation (Li and Chiang, 2012; Andersen et al., 2013; Ngan et al., 2014; Elsherif et al., 2017).

#### 3.1.2 Effect of the studied factors on particle size (Y_2_)

The PS and PDI for all prepared samples were measured and are presented in Table 2. PDI assesses the uniformity of particle distribution, while PS indicates the average hydrodynamic diameter of the particles (Das et al., 2012; ElMeshad and Mohsen, 2014; Al-Mahallawi et al., 2015; Shah et al., 2021). Formulations T1, T3, T4, and T6 exhibited high PDIs (over 0.5), suggesting these were more heterogeneous with less uniform particle sizes (ElMeshad and Mohsen, 2014; Albash et al., 2021a, Albash et al., 2021b; Shah et al., 2021) (data not shown). The high PS in these samples prompted the use of probe sonication.

Without probe sonication, particles in samples T1 to T6 were in the micro-range ranging from 1.619 ± 72 to 4.981 ± 101 μm. Therefore, probe sonication was applied to samples T7 to T12 to reduce the PS of the pectin nanoparticles, aiming for enhanced penetration during treatment application (Elsherif et al., 2017), and the particles were reduced to range from 0.493 ± 27 to 0.942 ± 31 μm. ANOVA results showed that only one factor, X_3_ (probe sonication time) had a significant impact on the PS of the NBV-loaded-pectin-NP (*p* < 0.0001). Increasing the probe sonication time (X_3_) led to a significant decrease in PS (Fig. 1C). This reduction was anticipated due to the ultrasonic radiation dispersing the particles into smaller sizes. These findings align with other researches (Li and Chiang, 2012; Ngan et al., 2014), and correlate with the EE % results, suggesting that NBV leakage from the particles is a consequence of disruption and reaggregation into smaller sizes.

#### 3.1.3 Effect of the studied factors on zeta potential (Y_3_)

Zeta potential is crucial for assessing the surface charge and stability of pectin nanoparticles (Cheng and Lim, 2004) (Chinnaiyan et al., 2018; Albash et al., 2021a, Albash et al., 2021b). A higher absolute ZP value, irrespective of its sign, indicates greater stability and reduced particle interactions. The ZP results ranged between −51.5 ± 0.21 and − 31.4 ± 0.07 mV as presented in Table 2. ANOVA results showed that both pectin to TPP ratio (X_1_) and pectin amount (X_2_) had significant effects on ZP (*p* < 0.0001 for both).

**Fig. 2:**
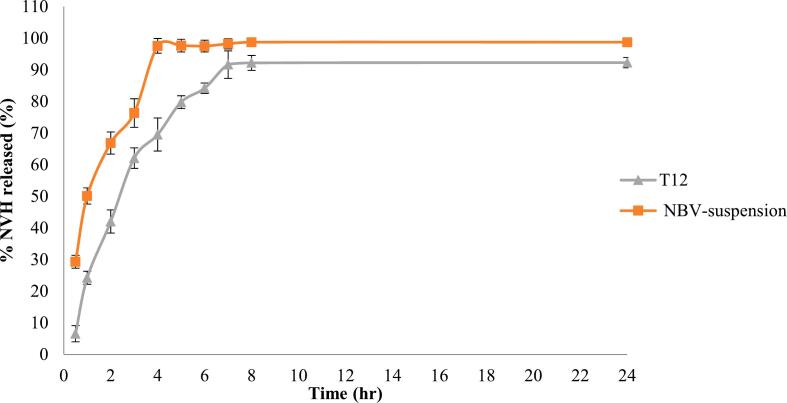
Release profile of NBV from a) the optimized NBV–loaded-pectin nanoparticles (T12) and b) NBV aqueous suspension. Abbreviations: NBV: nebivolol hydrochloride.

A notable pattern was observed, increasing the TPP amount from 1:0.075 to 1:0.15 led to a decrease in ZP, followed by an increase when TPP was further increased to 1:0.3 (Fig. 2D). These changes in ZP correspond well with EE % changes. At a low TPP level (0.075), insufficient TPP for crosslinking left excess pectin un-crosslinked, resulting in a high negative ZP value (Dogan Ergin et al., 2021). Conversely, at a TPP level of 0.15, the ZP and EE % both decreased, suggesting efficient crosslinking of TPP with available pectin, which increased particle rigidity and reduced encapsulation capacity. At a higher TPP level (0.3), both ZP and EE % increased again. This implies that the surplus TPP, being anionic, contributed to the rise in ZP value (Silvestro et al., 2020), suggesting more efficient encapsulation at this concentration.

Additionally, as shown in Fig. 1E, increasing the pectin amount from 50 mg to 100 mg led to a significant increase in ZP. This increase is likely due to the higher concentration of unesterified galacturonic acid, which occurs when more pectin is added to the preparation. The pectin used has an esterification degree of 63–66%, which enhances the negative charge of the TPP, thereby contributing to the observed elevation in ZP values. Similar results were observed by Dogan Ergin *et al* (Dogan Ergin et al., 2021) who reported similar shifts in negative charge (−10.9 to −25.5 mV) in their study on pectin nanoparticles in microparticles, using pectin with 55–70% esterification. They attributed these changes to the unesterified galacturonic acid chains in the pectin molecule.

Based on the data presented and considering the selection parameters outlined in Table 1, the formulation chosen using Design Expert® software was the NBV-loaded-pectin-NP T12. This selection was made due to its highest desirability value of 0.859, making it the optimal candidate for subsequent studies

### 3.2 Characterization of the optimized NBV-loaded-pectin-NP

#### 3.2.1 *In-vitro* release study

This study was conducted to evaluate the release profile of NBV from the optimized formulation T12 and compare its profile to the dissolution profile of NBV from an NBV aqueous suspension. As illustrated in Fig. 2, NBV dissolution from the aqueous suspension was rapid, with approximately 30% of the drug dissolving in the first 0.5 h. On the other hand, the optimal T12 formulation exhibited a more gradual release of the drug, with only 6.5% of NBV being released within 0.5 h. This could be attributed to the entrapment of NBV within the pectin chains of the nanoparticles, leading to a slower drug release (Pandit et al., 2017; Chinnaiyan et al., 2018).

Korsmeyer-Peppas model fitting of the release data suggested that NBV is released from the nanoparticles primarily through diffusion. Pectin, a widely used carrier in sustained and controlled oral drug delivery systems, modulates the release rate by controlling the swelling degree through osmotic effects (Sriamornsak et al., 2007). Rashidipour et al. (Rashidipour et al., 2019) indicated that the release of paraquet was by diffusion and swelling-controlled mechanisms. Similar to our findings, they reported minimal drug release in the first 0.5 h, attributing it to the time needed for pectin to swell and form a viscous gel in the aqueous release media. They also noted that the delayed release of paraquet was due to affinity-controlled mechanisms involving ionic interactions between pectin and the oppositely charged paraquat, similar to the interactions in our study between oppositely charged pectin and NBV (Guilherme et al., 2015). Additionally, Chinnaiyan *et al* (Chinnaiyan et al., 2018) confirmed a gradual release of metformin hydrochloride from pectin nanoparticles, which they attributed to the saturation of metformin within the biopolymer chains.

#### 3.2.2 Transmission electron microscopy (TEM)

**Fig. 3:**
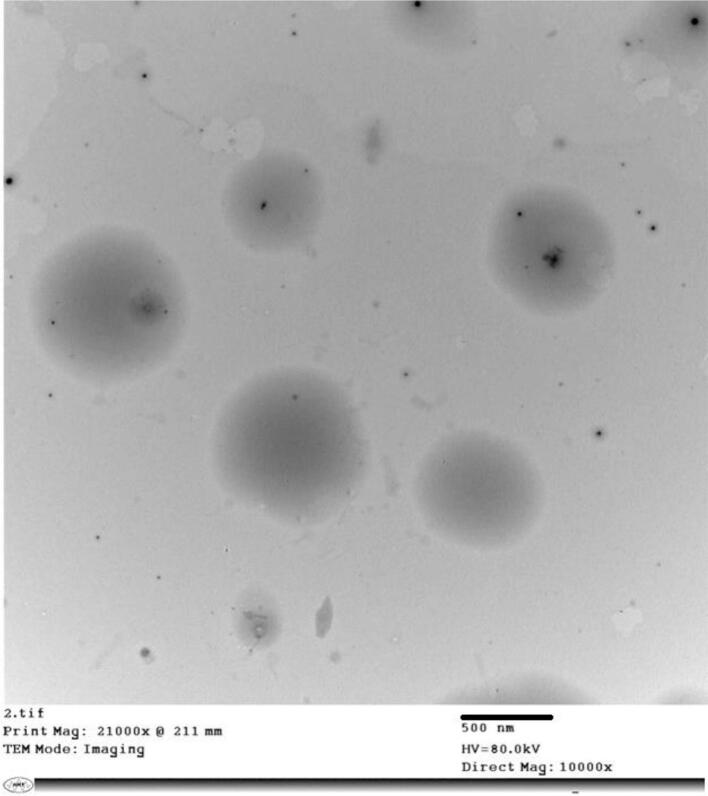
TEM photomicrograph of the optimized NBV–loaded pectin nanoparticles (T12).

The optimized T12 formulation was morphologically examined using TEM, and the photomicrograph is represented in Fig. 3. The TEM micrograph revealed that the optimal T12 formulation exhibited a rather spherical shape, with a size in accordance with that obtained from the zeta sizer analysis ranging from 0.5 to 0.7 μm.

#### 3.2.3 Differential Scanning Calorimetry (DSC)

To confirm any alteration in the state of NBV upon encapsulation within the optimized NBV-loaded-pectin-NP (T12) and to rule out potential interactions with other ingredients, a DSC study was conducted (Basha et al., 2013; Burapapadh et al., 2016; Üstündağ-Okur et al., 2016).

**Fig. 4:**
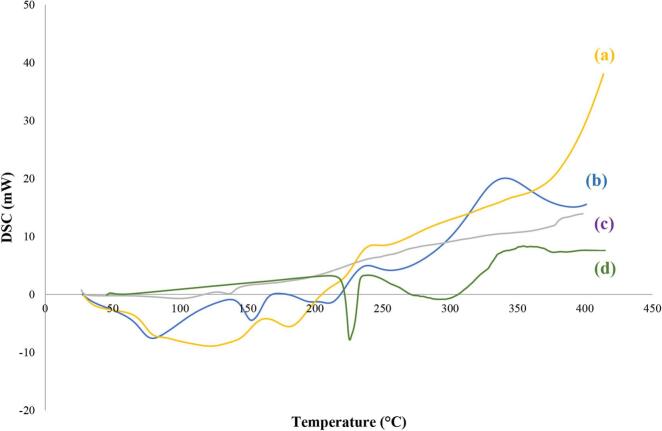
DSC thermograms of (a) Physical mixture of NBV and pectin (1:10 *w/w ratio*); (b) Pectin; (c) Lyophilized optimized NBV-loaded-pectin nanoparticles (T12); and (d) NBV. Abbreviations: DSC: Differential scanning calorimetry, NBV: nebivolol hydrochloride.

This study encompassed NBV, pectin, a physical mixture of NBV and pectin (1:10 ratio), and the lyophilized optimized T12 formulation. The results are illustrated in Fig. 4.

The DSC scan of NBV powder showed an endothermic peak at 228 °C, corresponding to the melting point of NBV, as previously reported (Patil, 2016; Sipos et al., 2016). The thermogram of pectin powder exhibited two broad endothermic peaks at 79 °C and 158 °C, corresponding to the glass transition temperature and melting point of pectin, respectively, in agreement with previous reports (Mittal and Kaur, 2014; Kala et al., 2020). In the physical mixture of NBV and pectin, there was a notable decrease in the intensity of the NBV peak and a reduction and broadening of the pectin peaks, likely due to the dilution effect.

The thermogram of the lyophilized optimized T12 formulation displayed a complete absence of characteristic peaks for both NBV and pectin. This could be attributed to encapsulation and dispersion of NBV in the T12 pectin nanoparticles (Barakat and Almurshedi, 2011; Rashidipour et al., 2019), suggesting the transformation of NBV to an amorphous form. These results are in agreement with Maged *et al* (Maged et al., 2019), where the DSC thermograms of their selected scaffolds revealed the disappearance of characteristic peaks of chitosan hydrochloride and carboxymethyl cellulose, attributed to a crosslinking complex formation among the ingredients used.

#### 3.2.4 X-ray diffractometry (XRD)

**Fig. 5:**
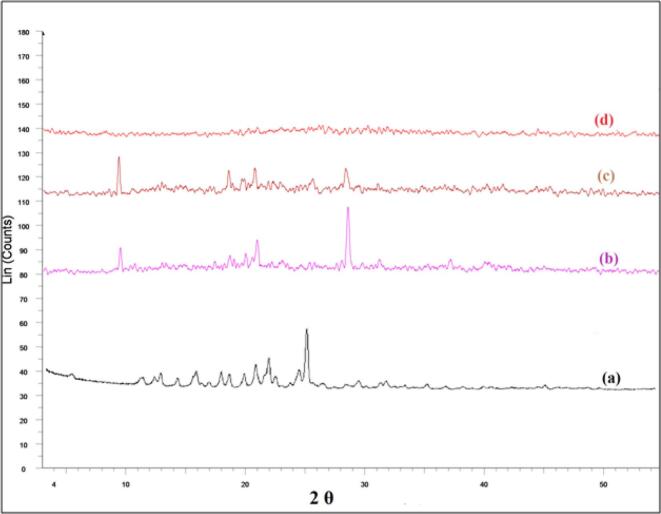
X-ray diffractograms of (a) NBV; (b) Pectin; (c) Physical mixture of NBV to pectin (1:10 *w/w ratio*); and (d) Lyophilized optimized NBV-loaded pectin nanoparticles (T12). Abbreviations: NBV: nebivolol hydrochloride.

To assess the impact of the method of preparation on the physical state of NBV, an XRD study was conducted (Pravala et al., 2013; Hanif et al., 2018). Fig. 5 illustrates the X-ray diffractograms of NBV, pectin, NBV: pectin physical mixture (1:10 ratio) and the optimized T12 nanoparticles. NBV exhibited distinct sharp peaks at 2θ values of 5.9°, 11.9°, 12.2°, 16.3°, 18.4°, 21.4°, 22.4°, and 25.67° (Fig. 5a), confirming its crystalline nature (Pravala et al., 2013; Hanif et al., 2018; Linga Reddy, 2019). On the other hand, pectin exhibited characteristic sharp peaks at 9.5°, 18.8°, 20.2°, 21.3° and 28.7° (Fig. 5b), aligning with previously reported data (Mishra et al., 2008, Mishra et al., 2011). The X-ray spectra of the physical mixture, as shown in Fig. 5c, revealed the disappearance of NBV peaks and a reduction in the intensity of pectin peaks. This observation is consistent with the dilution effect of pectin on NBV and supports the DSC results.

The diffractogram of T12 nanoparticles, presented in Fig. 5d, showed broadened peaks and the absence of sharp intensities of NBV, indicating the encapsulation of NBV in an amorphous form within the optimized pectin nanoparticles (Bagliotti Meneguin et al., 2014; Pandey et al., 2021). Additionally, the sharp peaks of pectin also disappeared which might be attributed to its crosslinking with TPP, leading to a change in its physical form.

### 3.3 *In vivo* animal studies

#### 3.3.1 Sterilization of the samples

Sterilization of the pectin nanoparticles was effectively achieved using gamma radiation, chosen for its benefit as a cold method, thereby minimizing potential damage to the samples (Silindir Gunay and Ozer, 2009; Fairand, 2001). A low radiation intensity of 10 kGy, applied at a rate of approximately 1 kGy/h (Maged et al., 2019; Morsi et al., 2019), was utilized to preserve the physio-chemical properties of the nanoparticles (Yang et al., 2002; Desai and Park, 2006; Kim et al., 2007; Morsi et al., 2019). To verify the safety of this radiation intensity, the characteristics of the optimized T12 nanoparticles were evaluated post-sterilization by measuring the PS and EE %. Results revealed no significant changes in PS and EE % (*p* ≤ 0.05) confirming the efficacy and safety of the method (results not shown).

#### 3.3.2 Assessment of wound healing

The excisional wound model was selected for this study as it closely simulates acute clinical wounds, allowing for detailed monitoring and analysis of the typical stages of wound healing (Masson-Meyers et al., 2020). Its relative simplicity and practicality further contributed to its selection as the preferred method in our study (Peplow et al., 2010; Wong et al., 2011).

In this study, the optimized T12 nanoparticles were not incorporated into a specific dosage form. This is because following separation *via* cooling centrifugation, the precipitated nanoparticles were in a gel-like state, which allowed their direct topical application to the wounds. Throughout the study, the wounds were examined daily, and no signs of inflammation, blood or puss were noted in any of the treatment groups.

**Fig. 6:**
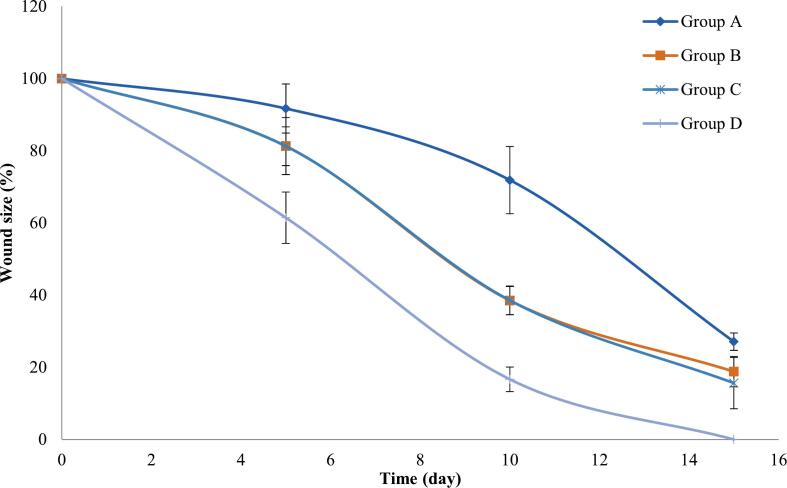
Analysis of wound size reduction over 15 days among the four treatment groups. Group A: the control group and received no treatment; Group B: received the NBV aqueous suspension; Group C: received the blank optimized pectin nanoparticles, Group D: received the optimized NBV-loaded pectin nanoparticles (T12). Abbreviations: NBV: nebivolol hydrochloride.

**Fig. 7:**
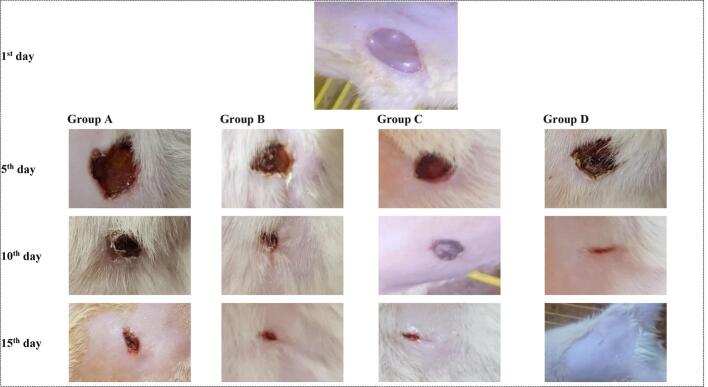
Sequential photographic comparison of wound healing progression at days 1, 5, 10, and 15 across the four treatment groups. Group A: the control group and received no treatment; Group B: received the NBV aqueous suspension; Group C: received the blank optimized pectin nanoparticles, Group D: received the optimized NBV-loaded pectin nanoparticles (T12). Abbreviations: NBV: nebivolol hydrochloride.

To monitor and compare wound healing progress, the wound sizes were measured and photographed on the 5th, 10th, and 15th days. The results are graphically presented in Fig. 6, with corresponding photographic images in Fig. 7. It was observed that by the 15th day, the hair had re-grown and obscured the wounds, and thus we had to re-shave the animals in all groups to present the changes clearly in the photos. Moreover, this was a necessary step prior to the histopathological study, thus it was conducted. The study duration was 15 days, as by which time the group treated with T12 nanoparticles (Group D) achieved complete wound closure. In contrast, the wound sizes in Group A (the untreated control group), Group B (the NBV aqueous suspension-treated group), and Group C (the blank pectin nanoparticles-treated group) were reduced to 27.1% ± 2.4%, 18.8% ± 4.2%, and 15.6% ± 7.1% of their initial sizes respectively. Statistical analysis revealed that Group D exhibited a non-significant greater reduction in wound size compared to the other groups on the 5th day. However, these differences became significantly more pronounced by the 10th day compared to Group A (*p* = 3.9 × 10^−6^) and Groups B and C (*p* = 0.002 for both). By day 15, this trend continued with Group D showing significantly better wound closure compared to Group A (*p* = 4.7 × 10^−8^), Group B (*p* = 2.58 × 10^−6^), and Group C (*p* = 0.0009). Additionally, Groups B and C showed very similar rates of wound contraction, particularly on the 5th and 10th days. By the 15th day, Group C demonstrated better wound closure compared to Group B, underscoring the synergistic effect of using NBV and pectin in combination, as opposed to their individual applications.

In the field of biomedical research, tissue regeneration and wound healing are crucial areas of study, particularly for conditions like diabetes where healing is often impaired. Nebivolol hydrochloride (NBV), a drug known for its vasodilator effects, and pectin, a natural polysaccharide, have shown promise in this regard.

Metineren *et al* (Metineren et al., 2017) studied the effect of intraperitoneal administration of NBV on fracture healing in rats. Their results suggested that NBV had a positive effect on fracture healing through the NO pathway and its direct vasodilator effects. Similarly, Pandit *et al* (Pandit et al., 2017) observed accelerated wound healing in diabetic rats treated with NBV-loaded microsponge gels, attributing this to improved fibroblasts and collagen activity at the wound site. Lee *et al* (Lee et al., 2019) further supported this by demonstrating how NO-releasing hydrogel dressings promoted wound healing in mice.

On the other hand, pectin has been recently introduced for wound healing preparations. In 2017, Giusto *et al* (Giusto et al., 2017) reported faster wound healing in rats with a pectin-honey hydrogel compared to honey alone, underscoring pectin's effectiveness. In 2020, Zulema *et al* (Zulema et al., 2020) reinforced this with their study on a pectin-allantoin film, which significantly expedited wound closure in rats.

#### 3.3.3 Histopathological study

**Fig. 8:**
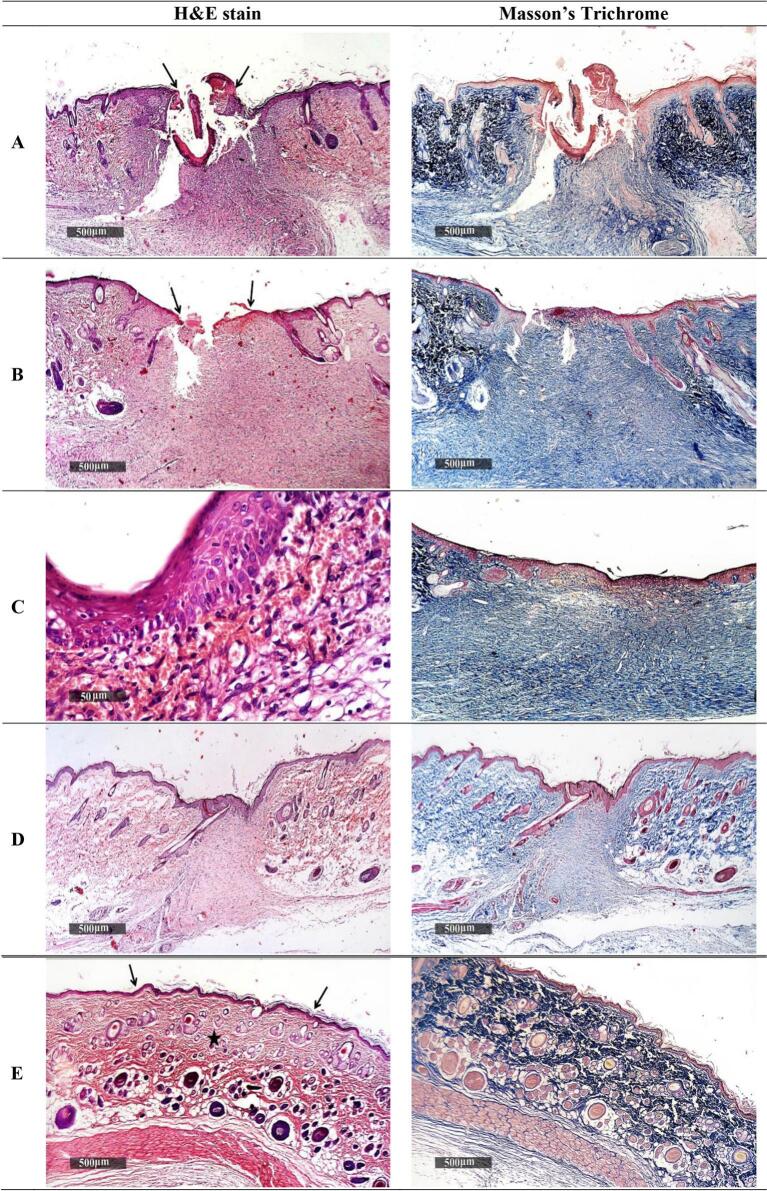
Microscopic histopathological sections showcasing the differences in tissue response across five distinct groups. Group A represents the non-treated control, exhibiting the natural wound healing process. Group B shows the effects of treatment with an NBV aqueous suspension. Group C displays the response to treatment with blank pectin nanoparticles. Group D illustrates the impact of treatment with the optimized NBV-loaded-pectin nanoparticles (formulation T12). Group E provides a comparison with normal, uninjured skin tissue, serving as a baseline negative control. Each section reveals key differences in cellular and tissue structures, reflecting the varying degrees of wound healing and tissue regeneration among the groups. Abbreviations: NBV: Nebivolol hydrochloride.

This study involved examining histopathological sections from wound sites across the four treatment-groups, stained with Hematoxylin and Eosin (H&E) and Masson's trichrome. These sections, assessed 15 days after inducing wounds, were examined under a light microscope. The observations, alongside a normal control skin sample (negative control), are presented in Fig. 8. The following results were obtained.

Group A (untreated): The skin in this group displayed continuous loss of the epidermal layer and ulceration, characterized by subepidermal hemorrhagic patches and a high concentration of cellular granulation tissue rich in inflammatory cells (Fig. 8A). Moreover, the dermal collagen deposition was disorganized and significantly reduced (*p* = 2.28 × 10^−13^) compared to normal skin sample, indicating deficient wound healing (Maged et al., 2019).

Group B (NBV aqueous suspension): This group showed persistent, localized loss of the epidermal layer, along with subepidermal hemorrhagic patches and partial re-epithelialization. There was also cellular granulation tissue with mild infiltration of inflammatory cells (Fig. 8B). The accelerated proliferation observed here could be attributed to the presence of NBV, likely due to an increased NO levels promoting vasodilatation and thereby enhancing wound closure and collagen synthesis (Pandit et al., 2017). Furthermore, minimal collagen fibers formation was observed, yet significantly (*p* = 5.25 × 10^−8^) lower than the negative control normal skin (sample E).

Group C (pectin nanoparticles without NBV): In this group, complete wound closure was noted, as evidenced by the re-epithelialization of the epidermal layer and the presence of congested and dilated subepidermal blood vessels (Fig. 8C). Mature collagen bundles presented by the circular shapes were available but still significantly less than those of the negative control normal skin (sample E) (*p* = 3.2 × 10^−6^).

Group D (T12 nanoparticles): Sections from this group demonstrated complete healing of the epidermal layer with the formation of new keratinocytes and mild, persistent dermal granulation tissue (Fig. 8D). Furthermore, the wound area was enriched with fibrous granulation tissue and mature collagen bundles, whose levels were statistically similar (*p* = 0.146) to the negative control group (Fig. 8E), demonstrating the dual synergistic effect of both NBV and pectin on wound healing. Similar results were observed by Lee *et al* (Lee et al., 2019) upon the application of NO releasing hydrogel on infected mice wounds after 14 days of the initiation of drug treatment.

These histopathological observations were in accordance with the wound contraction measurement results and the macroscopic images of the healing progress of wounds.

In summary, these studies collectively highlight the potential of NBV and pectin in wound healing applications. The vasodilator and NO pathway effects of NBV, alongside pectin's properties as a biopolymer, offer promising avenues for future research, especially in developing more effective treatments for impaired wound healing. While these findings are encouraging, further research is needed to fully understand the long-term effects and efficacy across different wound types.

## 4 Conclusion

In this study, we successfully utilized the ionotropic gelation method to develop Nebivolol (NBV)-loaded pectin nanoparticles, guided by a 2^2^3^1^ full factorial design. The optimization process, facilitated by Design expert® software, led us to select an optimal formulation (T12) that exhibited high encapsulation efficiency (EE %) and Zeta potential (ZP), along with small, spherical, non-aggregated particle size (PS). Solid-state characterization confirmed the amorphous dispersion of NBV within these nanoparticles, indicating effective drug incorporation.

The *in vivo* animal study highlighted a potential dual synergistic action of NBV and pectin, which significantly accelerated the wound healing process. These findings are promising, suggesting that the optimized NBV-loaded-pectin-NP not only serves as an efficient wound healing accelerator but also acts as a potent tissue regenerator. This approach might offer a new avenue in advanced wound care, potentially improving outcomes in tissue repair and regeneration.

## Funding

This research did not receive any specific grant from funding agencies in the public, commercial, or not-for-profit sectors.

## Institutional review board statement

All animal studies were performed following the protocol approved by the Research Ethics Committee (REC, PI 1965, 27 April 2017) of the Faculty of Pharmacy, Cairo University, Cairo, Egypt. These studies were in compliance with the guidelines and regulations of the international guiding principles for the use of animals in biomedical research.

## Informed consent statement

No human subjects were involved in this study.

## CRediT authorship contribution statement

**Noha I. Elsherif:** Writing – original draft, Software, Resources, Methodology, Investigation, Formal analysis. **Abdulaziz M. Al-Mahallawi:** Software, Supervision, Writing – review & editing. **Iman Saad Ahmed:** Writing – original draft, Resources. **Rehab N. Shamma:** Writing – review & editing, Software, Methodology, Investigation, Formal analysis.

## Declaration of competing interest

The authors declare that they have no known competing financial interests or personal relationships that could have appeared to influence the work reported in this paper.

## Data Availability

Data available upon request.

## References

[bb0005] Abdelbary A., AbouGhaly M.H. (2015). Design and optimization of topical methotrexate loaded niosomes for enhanced management of psoriasis: application of Box-Behnken design, in-vitro evaluation and in-vivo skin deposition study. Int. J. Pharm..

[bb0010] Abdelbary A., Abd-Elsalam W.H., Al-Mahallawi A.M. (2019). Fabrication of levofloxacin polyethylene glycol decorated nanoliposomes for enhanced management of acute otitis media: Statistical optimization, trans-tympanic permeation and in vivo evaluation. Int. J. Pharm..

[bb0015] Ahmed M., Al-mahallawi A.M., El-Helaly S.N., Abd-Elsalam W.H. (2019). The effect of the saturation degree of phospholipid on the formation of a novel self-assembled nano-micellar complex carrier with enhanced intestinal permeability. Int. J. Pharm..

[bb0020] Ahmed R., Augustine R., Chaudhry M., Akhtar U.A., Zahid A.A., Tariq M., Falahati M., Ahmad I.S., Hasan A. (2022). Nitric oxide-releasing biomaterials for promoting wound healing in impaired diabetic wounds: state of the art and recent trends. Biomed. Pharmacother..

[bb0025] Albash R., Al-Mahallawi A.M., Hassan M., Alaa-Eldin A.A. (2021). Development and optimization of terpene-enriched vesicles (Terpesomes) for effective ocular delivery of fenticonazole nitrate: in vitro characterization and in vivo assessment. Int. J. Nanomedicine.

[bb0030] Albash R., Yousry C., Al-Mahallawi A.M., Alaa-Eldin A.A. (2021). Utilization of PEGylated cerosomes for effective topical delivery of fenticonazole nitrate: in-vitro characterization, statistical optimization, and in-vivo assessment. Drug Deliv..

[bb0035] Al-Dhubiab B., Nair A., Kumria R., Attimarad M., Harsha S. (2019). Development and evaluation of nebivolol hydrochloride nanocrystals impregnated buccal film. Farmacia.

[bb0040] Alipour R., Khorshidi A., Shojaei A., Mashayekhi F., Moghaddam M.J.M. (2019). Skin wound healing acceleration by Ag nanoparticles embedded in PVA/PVP/Pectin/Mafenide acetate composite nanofibers. Polym. Test..

[bb0045] Al-Mahallawi A., Abdelbary A.A., Aburahma M.H. (2015). Investigating the potential of employing bilosomes as a novel vesicular carrier for transdermal delivery of tenoxicam. Int. J. Pharm..

[bb0050] Al-Mahallawi A., Abdelbary A.A., El-Zahaby S.A. (2021). Norfloxacin loaded nano-cubosomes for enhanced management of otitis externa: in vitro and in vivo evaluation. Int. J. Pharm..

[bb0055] Al-mahallawi A., Ahmed D., Hassan M., El-Setouhy D.A. (2021). Enhanced ocular delivery of clotrimazole via loading into mucoadhesive microemulsion system: in vitro characterization and in vivo assessment. J. Drug. Deliv. Sci. Tech..

[bb0060] Alvarez-Lorenzo C., Blanco-Fernandez B., Puga A.M., Concheiro A. (2013). Crosslinked ionic polysaccharides for stimuli-sensitive drug delivery. Adv. Drug Deliv. Rev..

[bb0065] Amrutiya N., Bajaj A., Madan M. (2009). Development of microsponges for topical delivery of mupirocin. AAPS PharmSciTech.

[bb0070] Andersen T., Vanić Ž., Flaten G.E., Mattsson S., Tho I., Škalko-Basnet N. (2013). Pectosomes and chitosomes as delivery systems for metronidazole: the one-pot preparation method. Pharmaceutics.

[bb0075] Andriotis E., Eleftheriadis G.K., Karavasili C., Fatouros D.G. (2020). Development of bio-active patches based on pectin for the treatment of ulcers and wounds using 3D-bioprinting technology. Pharmaceutics.

[bb0080] Auriemma G., Russo P., Del Gaudio P., García-González C.A., Landín M., Aquino R.P. (2020). Technologies and formulation design of polysaccharide-based hydrogels for drug delivery. Molecules.

[bb0085] Bagliotti Meneguin A., Stringhetti Ferreira Cury B., Evangelista R.C. (2014). Films from resistant starch-pectin dispersions intended for colonic drug delivery. Carbohydr. Polym..

[bb0090] Barakat N., Almurshedi A.S. (2011). Design and development of gliclazide-loaded chitosan microparticles for oral sustained drug delivery: in-vitro/in-vivo evaluation. J. Pharm. Pharmacol..

[bb0095] Basha M., Abd El-Alim S.H., Shamma R.N., Awad G.E. (2013). Design and optimization of surfactant-based nanovesicles for ocular delivery of Clotrimazole. J. Liposome Res..

[bb0100] Birch N., Schiffman J.D. (2014). Characterization of self-assembled polyelectrolyte complex nanoparticles formed from chitosan and pectin. Langmuir.

[bb0105] Bostancı N., Büyüksungur S., Hasirci N., Tezcaner A. (2022). Potential of pectin for biomedical applications: a comprehensive review. J. Biomater. Sci. Polym. Ed..

[bb0110] Burapapadh K., Takeuchi H., Sriamornsak P. (2016). Development of pectin nanoparticles through mechanical homogenization for dissolution enhancement of itraconazole. Asian J. Pharm. Sci..

[bb0115] Calvo P., Remuñan-López C., Vila-Jato J.L., Alonso M.J. (1997). Chitosan and chitosan/ethylene oxide-propylene oxide block copolymer nanoparticles as novel carriers for proteins and vaccines. Pharm. Res..

[bb0120] Cao L., Lu W., Mata A., Nishinari K., Fang Y. (2020). Egg-box model-based gelation of alginate and pectin: a review. Carbohydr. Polym..

[bb0125] Cheng K., Lim L.Y. (2004). Insulin-loaded calcium pectinate nanoparticles: effects of pectin molecular weight and formulation pH. Drug Dev. Ind. Pharm..

[bb0130] Chinnaiyan S., Karthikeyan D., Gadela V.R. (2018). Development and characterization of metformin loaded pectin nanoparticles for T2 diabetes mellitus. Pharm Nanotechnol..

[bb0135] Culling C. (2013).

[bb0140] Das S., Ng W.K., Tan R.B. (2012). Are nanostructured lipid carriers (NLCs) better than solid lipid nanoparticles (SLNs): development, characterizations and comparative evaluations of clotrimazole-loaded SLNs and NLCs?. Eur. J. Pharm. Sci..

[bb0145] Desai K., Park H.J. (2006). Study of gamma-irradiation effects on chitosan microparticles. Drug Deliv..

[bb0150] Devasvaran K., Lim V. (2021). Green synthesis of metallic nanoparticles using pectin as a reducing agent: a systematic review of the biological activities. Pharm. Biol..

[bb0155] Dogan Ergin A., Bayindir Z.S., Ozcelikay A.T., Yuksel N. (2021). A novel delivery system for enhancing bioavailability of S-adenosyl-l-methionine: Pectin nanoparticles-in-microparticles and their in vitro - in vivo evaluation. J. Drug. Deliv. Sci. Tech..

[bb0160] El Said H., Lalatsa A., Al-Mahallawi A.M., Saddar El Leithy E., Ghorab D.M. (2022). Vilazodone-phospholipid mixed micelles for enhancing oral bioavailability and reducing pharmacokinetic variability between fed and fasted states. Int. J. Pharm..

[bb0165] El-Bahy A., Aboulmagd Y.M., Zaki M. (2018). Diabetex: a novel approach for diabetic wound healing. Life Sci..

[bb0170] ElMeshad A., Mohsen A.M. (2014). Enhanced corneal permeation and antimycotic activity of itraconazole against *Candida albicans* via a novel nanosystem vesicle. Drug Deliv..

[bb0175] Elsherif N., Shamma R.N., Abdelbary G. (2017). Terbinafine hydrochloride trans-ungual delivery via nanovesicular systems: in vitro characterization and ex vivo evaluation. AAPS PharmSciTech.

[bb0180] Elsherif N., Al-Mahallawi A.M., Abdelkhalek A.A., Shamma R.N. (2021). Investigation of the potential of nebivolol hydrochloride-loaded chitosomal systems for tissue regeneration: in vitro characterization and in vivo assessment. Pharmaceutics.

[bb0185] Fahmy A., Hassan M., El-Setouhy D.A., Tayel S.A., Al-Mahallawi A.M. (2020). Voriconazole ternary micellar systems for the treatment of ocular mycosis: statistical optimization and in vivo evaluation. J. Pharm. Sci..

[bb0190] Fahmy A., Hassan M., El-Setouhy D.A., Tayel S.A., Al-Mahallawi A.M. (2021). Statistical optimization of hyaluronic acid enriched ultradeformable elastosomes for ocular delivery of voriconazole via Box-Behnken design: in vitro characterization and in vivo evaluation. Drug Deliv..

[bb0195] Fairand B. (2001).

[bb0200] Farahnaky A., Sharifi S., Imani B., Dorodmand M.M., Majzoobi M. (2018). Physicochemical and mechanical properties of pectin-carbon nanotubes films produced by chemical bonding. Food Packag. Shelf Life.

[bb0205] Frank S., Kämpfer H., Wetzler C., Pfeilschifter J. (2002). Nitric oxide drives skin repair: Novel functions of an established mediator. Kidney Int..

[bb0210] Friedman A., Han G., Navati M.S., Chacko M., Gunther L., Alfieri A., Friedman J.M. (2008). Sustained release nitric oxide releasing nanoparticles: characterization of a novel delivery platform based on nitrite containing hydrogel/glass composites. Nitric Oxide.

[bb0215] Giusto G., Vercelli C., Comino F., Caramello V., Tursi M., Gandini M. (2017). A new, easy-to-make pectin-honey hydrogel enhances wound healing in rats. BMC Complement. Altern. Med..

[bb0220] Grant G., Morris E.R., Rees D.A., Smith P.J.C., Thom D. (1973). Biological interactions between polysaccharides and divalent cations: the Egg-box model. FEBS Lett..

[bb0225] Guilherme M., Aouada F.A., Fajardo A.R., Martins A.F., Paulino A.T., Davi M.F.T., Rubira A.F., Muniz E.C. (2015). Superabsorbent hydrogels based on polysaccharides for application in agriculture as soil conditioner and nutrient carrier: a review. Eur. Polym. J..

[bb0230] Gulcan E., Kuçuk A., Çayci K., Tosun M., Emre H., Koral L., Aktan Y., Avsar U. (2012). Topical effects of nebivolol on wounds in diabetic rats. Eur. J. Pharm. Sci..

[bb0235] Hanif M., Khan H., Afzal S., Mahmood D., Maheen S., Afzal K., Iqbal N., Andleeb M., Abbas N. (2018). Sustained release biodegradable solid lipid microparticles: Formulation, evaluation and statistical optimization by response surface methodology. Acta Pharma..

[bb0240] Hanif M., Khan H., Afzal S., Majeed A., Iqbal N., Afzal K., Andleeb M., Rauf A., Farooq A. (2019). Formulation, characterization and optimization of nebivolol-loaded sustained release lipospheres. Trop. J. Pharm. Res..

[bb0245] Hilas O., Ezzo D. (2009). Nebivolol (bystolic), a novel Beta blocker for hypertension. Pharm Ther.

[bb0250] Jacob E., Borah A., Jindal A., Pillai S.C., Yamamoto Y., Maekawa T., Kumar D.N.S. (2020). Synthesis and characterization of citrus-derived pectin nanoparticles based on their degree of esterification. J. Mater. Res..

[bb0255] Jatav V., Saggu J.S., Sharma A.K., Sharma A., Jat R.K. (2013). Design, development and permeation studies of nebivolol hydrochloride from novel matrix type transdermal patches. Adv. Biomed. Res..

[bb0260] Jonassen H., Treves A., Kjøniksen A.L., Smistad G., Hiorth M. (2013). Preparation of ionically cross-linked pectin nanoparticles in the presence of chlorides of divalent and monovalent cations. Biomacromolecules.

[bb0265] Jones M., Ganopolsky J.G., Labbé A., Prakash S. (2010). A novel nitric oxide producing probiotic patch and its antimicrobial efficacy: preparation and in vitro analysis. Appl. Microbiol. Biotechnol..

[bb0270] Kala S., Sogan N., Naik S.N., Agarwal A., Kumar J. (2020). Impregnation of pectin-cedarwood essential oil nanocapsules onto mini cotton bag improves larvicidal performances. Sci. Rep..

[bb0275] Kim I., Yoo M.K., Seo J.H., Park S.S., Na H.S., Lee H.C., Kim S.K., Cho C.S. (2007). Evaluation of semi-interpenetrating polymer networks composed of chitosan and poloxamer for wound dressing application. Int. J. Pharm..

[bb0280] Kumbhar D., Pokharkar V.B. (2013). Engineering of a nanostructured lipid carrier for the poorly water-soluble drug, bicalutamide: physicochemical investigations. Colloids Surf. A Physiochem..

[bb0285] Lee J., Hlaing S.P., Cao J., Hasan N., Ahn H.J., Song K.W., Yoo J.W. (2019). In situ hydrogel-forming/nitric oxide-releasing wound dressing for enhanced antibacterial activity and healing in mice with infected wounds. Pharmaceutics.

[bb0290] Li P., Chiang B.H. (2012). Process optimization and stability of D-limonene-in-water nanoemulsions prepared by ultrasonic emulsification using response surface methodology. Ultrason. Sonochem..

[bb0295] Linga Reddy B. (2019). X-Ray powder diffraction and crystallographic data of nebivolol hydrochloride. Int. J. Mater. Sci..

[bb0300] Luo J., Chen A. (2005). Nitric oxide: a newly discovered function on wound healing. Acta Pharmacol. Sin..

[bb0305] Maged A., Abdelkhalek A.A., Mahmoud A.A., Salah S., Ammar M.M., Ghorab M.M. (2019). Mesenchymal stem cells associated with chitosan scaffolds loaded with rosuvastatin to improve wound healing. Eur. J. Pharm. Sci..

[bb0310] Mamidi N., García R.G., Martínez J.D.H., Briones C.M., Martínez Ramos A.M., Tamez M.F.L., Del Valle B.G., Segura F.J.M. (2022). Recent advances in designing fibrous biomaterials for the domain of biomedical, clinical, and environmental applications. ACS Biomater Sci. Eng..

[bb0315] Mamidi N., Velasco Delgadillo R.M., Barrera E.V., Ramakrishna S., Annabi N. (2022). Carbonaceous nanomaterials incorporated biomaterials: the present and future of the flourishing field. Compos. Part B Eng..

[bb0320] Masson-Meyers D., Andrade T.A.M., Caetano G.F., Guimaraes F.R., Leite M.N., Leite S.N., Frade M.A.C. (2020). Experimental models and methods for cutaneous wound healing assessment. Int. J. Exp. Pathol..

[bb0330] Metineren H., Dülgeroğlu T.C., Metineren M.H., Aydın E. (2017). Effect of nebivolol on fracture healing: an experimental rat model. Adv. Clin. Exp. Med..

[bb0335] Meyyanathan S., Birajdar A., Suresh B. (2010). Simultaneous estimation of nebivolol hydrochloride and valsartan and nebivolol hydrochloride and hydrochlorothiazide in pharmaceutical formulations by UV spectrophotometric methods. Indian J. Pharma. Educ. Res..

[bb0340] Mishra R., Datt M., Banthia A.K. (2008). Synthesis and characterization of pectin/PVP hydrogel membranes for drug delivery system. AAPS PharmSciTech.

[bb0345] Mishra R., Majeed A., Banthia A. (2011). Development and characterization of pectin/gelatin hydrogel membranes for wound dressing. Int. J. Plast..

[bb0350] Mishra R., Banthia A., Majeed A. (2012). Pectin based formulations for biomedical applications: a review. Asian J. Pharm. Clin. Res..

[bb0355] Mittal N., Kaur G. (2014). In situ gelling ophthalmic drug delivery system: formulation and evaluation. J. Appl. Polym. Sci..

[bb0360] Mittal N., Mittal R. (2021). Repurposing old molecules for new indications: defining pillars of success from lessons in the past. Eur. J. Pharmacol..

[bb0365] Morsi N., Shamma R.N., Eladawy N.O., Abdelkhalek A.A. (2019). Bioactive injectable triple acting thermosensitive hydrogel enriched with nano-hydroxyapatite for bone regeneration: in-vitro characterization, Saos-2 cell line cell viability and osteogenic markers evaluation. Drug Dev. Ind. Pharm..

[bb0370] Nagar H., Srivastava A.K., Srivastava R., Kurmi M.L., Chandel H.S., Ranawat M.S. (2016). Pharmacological Investigation of the Wound Healing activity of *Cestrum nocturnum* (L.) Ointment in Wistar Albino Rats. Pharmaceutics.

[bb0375] Nemiwal M., Zhang T.C., Kumar D. (2021). Pectin modified metal nanoparticles and their application in property modification of biosensors. Carbohydr. Polym. Technol. Appl..

[bb0380] Ngan C., Basri M., Lye F.F., Fard Masoumi H.R., Tripathy M., Karjiban R.A., Abdul-Malek E. (2014). Comparison of process parameter optimization using different designs in nanoemulsion-based formulation for transdermal delivery of fullerene. Int. J. Nanomedicine.

[bb0385] Oveissi F., Tavakoli N., Minaiyan M., Mofid M.R., Taheri A. (2020). Alginate hydrogel enriched with Ambystoma mexicanum epidermal lipoxygenase-loaded pectin nanoparticles for enhanced wound healing. J. Biomater. Appl..

[bb0390] Pandey M., Choudhury H., Segar Singh S.K., Chetty Annan N., Bhattamisra S.K., Gorain B., Mohd Amin M.C.I. (2021). Budesonide-loaded pectin/polyacrylamide hydrogel for sustained delivery: fabrication, characterization and in vitro release kinetics. Molecules.

[bb0395] Pandit A., Patel S.A., Bhanushali V.P., Kulkarni V.S., Kakad V.D. (2017). Nebivolol-loaded microsponge gel for healing of diabetic wound. AAPS PharmSciTech.

[bb0400] Parker A., Boulenguer P., Kravtchenko T.P., Nishinari K., Doi E. (1993). Effect of the Addition of High Methoxy Pectin on the Rheology and Colloidal Stability of Acid Milk Drinks. Food Hydrocolloids: Structures, Properties, and Functions.

[bb0405] Patil J. (2016). Formulation, characterization and in vivo evaluation of novel edible dosage form containing nebivolol HCl. Braz. J. Pharm. Sci..

[bb0410] Patrulea V., Ostafe V., Borchard G., Jordan O. (2015). Chitosan as a starting material for wound healing applications. Eur. J. Pharm. Biopharm..

[bb0415] Peplow P., Chung T.Y., Baxter G.D. (2010). Laser photobiomodulation of proliferation of cells in culture: a review of human and animal studies. Photomed. Laser Surg..

[bb0420] Piao H., Ouyang M., Xia D., Quan P., Xiao W., Song Y., Cui F. (2011). In vitro-in vivo study of CoQ10-loaded lipid nanoparticles in comparison with nanocrystals. Int. J. Pharm..

[bb0425] Pinto R., Carvalho S., Antunes F., Pires J., Pinto M.L. (2022). Emerging nitric oxide and hydrogen sulfide releasing carriers for skin wound healing therapy. ChemMedChem.

[bb0430] Pistone S., Goycoolea F.M., Young A., Smistad G., Hiorth M. (2017). Formulation of polysaccharide-based nanoparticles for local administration into the oral cavity. Eur. J. Pharm. Sci..

[bb0435] Pramanik D., Ganguly M. (2018). Formulation and evaluation of a pectin based controlled drug delivery system containing metronidazole. Res. J. Life Sci..

[bb0440] Pravala K., Nagabandi V.K., Divya A. (2013). Enhancement of bioavailability of nebivolol hydrochloride through liquisolid formulations: in vitro and in vivo evaluation. Der. Pharm. Lett..

[bb0445] Rajapaksha D., Edirisinghe S., Nikapitiya C., Dananjaya S., Kwun H.J., Kim C.H., Oh C., Kang D.H., De Zoysa M. (2020). Spirulina maxima derived pectin nanoparticles enhance the immunomodulation, stress tolerance, and wound healing in zebrafish. Mar. Drugs.

[bb0450] Rao A., Rajeswari K.R., Sankar G.G. (2010). Spectrophotometric method for the determination of nebivolol hydrochloride in bulk and pharmaceutical formulations. Eur. J. Chem..

[bb0455] Rashidipour M., Maleki A., Kordi S., Birjandi M., Pajouhi N., Mohammadi E., Heydari R., Rezaee R., Rasoulian B., Davari B. (2019). Pectin/chitosan/tripolyphosphate nanoparticles: efficient carriers for reducing soil sorption, cytotoxicity, and mutagenicity of paraquat and enhancing its herbicide activity. J. Agric. Food Chem..

[bb0460] Ro J., Kim Y., Kim H., Park K., Lee K.E., Khadka P., Yun G., Park J., Chang S.T., Lee J., Jeong J.H., Lee J. (2015). Pectin micro- and nano-capsules of retinyl palmitate as cosmeceutical carriers for stabilized skin transport. Korean J. Physiol. Pharmacol..

[bb0465] Schaffer M., Tantry U., Gross S.S., Wasserburg H.L., Barbul A. (1996). Nitric oxide regulates wound healing. J. Surg. Res..

[bb0470] Schäffer M., Tantry U., Ahrendt G.M., Wasserkrug H.L., Barbul A. (1997). Acute protein-calorie malnutrition impairs wound healing: a possible role of decreased wound nitric oxide synthesis. J. Am. Coll. Surg..

[bb0475] Shah I., Bhatt S., Yadav A. (2014). Enhancement of solubility and dissolution of nebivolol by solid dispersion technique. Int J Pharm Pharm Sci.

[bb0480] Shah P., Chavda K., Vyas B., Patel S. (2021). Formulation development of linagliptin solid lipid nanoparticles for oral bioavailability enhancement: role of P-gp inhibition. Drug Deliv. Transl. Res..

[bb0490] Sharma M., Sharma R., Jain D.K. (2018). Preparation, characterization and evaluation of nebivolol loaded chitosan nanoparticles. J. Drug. Deliv. Ther..

[bb0495] Sharma R., Ahuja M., Kaur H. (2012). Thiolated pectin nanoparticles: Preparation, characterization and ex vivo corneal permeation study. Carbohydr. Polym..

[bb0500] Shekhter A., Serezhenkov V.A., Rudenko T.G., Pekshev A.V., Vanin A.F. (2005). Beneficial effect of gaseous nitric oxide on the healing of skin wounds. Nitric Oxide.

[bb0505] Shin J., Schoenfisch M.H. (2008). Inorganic/organic hybrid silica nanoparticles as a nitric oxide delivery scaffold. Chem. Mater..

[bb0510] Shrestha B., Haylor J. (2014). Experimental rat models of chronic allograft nephropathy: a review. Int. J. Nephrol. Renov. Dis..

[bb0515] Silindir Gunay M., Ozer Y. (2009). Sterilization methods and the comparison of E-Beam sterilization with gamma radiation sterilization. FABAD J. Pharm. Sci..

[bb0520] Silvestro I., Francolini I., Di Lisio V., Martinelli A., Pietrelli L., Scotto d’Abusco A., Scoppio A., Piozzi A. (2020). Preparation and characterization of TPP-Chitosan crosslinked scaffolds for tissue engineering. Materials.

[bb0525] Sipos E., Szabó Z.I., Redai E., Szabó P., Sebe I., Zelko R. (2016). Preparation and characterization of nanofibrous sheets for enhanced oral dissolution of nebivolol hydrochloride. J. Pharm. Biomed. Anal..

[bb0530] Sirohi B., Sagar R. (2019). Effect of hydroalcoholic extract of dactylorhiza hatagirea roots & lavandula stoechas flower on thiopental sodium induced hypnosis in mice. J. Drug. Deliv. Ther..

[bb0535] Sriamornsak P., Nunthanid J. (1999). Calcium pectinate gel beads for controlled release drug delivery: II. Effect of formulation and processing variables on drug release. J. Microencapsul..

[bb0540] Sriamornsak P., Thirawong N., Weerapol Y., Nunthanid J., Sungthongjeen S. (2007). Swelling and erosion of pectin matrix tablets and their impact on drug release behavior. Eur. J. Pharm. Biopharm..

[bb0545] Tavares G., Alves P., Simões P. (2022). Recent advances in Hydrogel-Mediated Nitric Oxide delivery Systems Targeted for Wound Healing applications. Pharmaceutics.

[bb0550] Tayel S., El-Nabarawi M.A., Tadros M.I., Abd-Elsalam W.H. (2015). Duodenum-triggered delivery of pravastatin sodium via enteric surface-coated nanovesicular spanlastic dispersions: development, characterization and pharmacokinetic assessments. Int. J. Pharm..

[bb0555] Thadkala K., Sailu C., Aukunuru J. (2015). Formulation, optimization and evaluation of oral nanosuspension tablets of nebivolol hydrochloride for enhancement of dissoluton rate. Der. Pharm. Lett..

[bb0560] Ulger B., Kapan M., Uslukaya O., Bozdag Z., Turkoglu A., Alabalık U., Onder A. (2016). Comparing the effects of nebivolol and dexpanthenol on wound healing: an experimental study. Int. Wound J..

[bb0565] Üstündağ-Okur N., Yurdasiper A., Gündoğdu E., Gökçe E.H. (2016). Modification of solid lipid nanoparticles loaded with nebivolol hydrochloride for improvement of oral bioavailability in treatment of hypertension: polyethylene glycol versus chitosan oligosaccharide lactate. J. Microencapsul..

[bb0570] Verma A., Chanchal A., Kumar A. (2011).

[bb0575] Voragen A., Coenen G.J., Verhoef R., Schols H.A. (2009). Pectin, a versatile polysaccharide present in plant cell walls. J. Struct. Chem..

[bb0580] Wakerley Z., Fell J.T., Attwood D., Parkins D.A. (1996). In vitro evaluation of pectin based colonic drug delivery systems. Int. J. Pharm..

[bb0585] Wang J., Plourde N.M., Iverson N., Moghe P.V., Uhrich K.E. (2007). Nanoscale amphiphilic macromolecules as lipoprotein inhibitors: the role of charge and architecture. Int. J. Nanomedicine.

[bb0590] Wong V., Sorkin M., Glotzbach J.P., Longaker M.T., Gurtner G.C. (2011). Surgical approaches to create murine models of human wound healing. J. Biomed. Biotechnol..

[bb0595] Yang F., Li X., Cheng M., Gong Y., Zhao N., Zhang X., Yang Y. (2002). Performance modification of chitosan membranes induced by gamma irradiation. J. Biomater. Appl..

[bb0600] Zulema K., Saucedo R., Ríos-Arana J., Lobo N., Rodriguez C., Cuevas-Gonzalez J., Tovar K. (2020). Natural Film based on Pectin and Allantoin for Wound Healing Obtaining, Characterization, and Rat Model. Biomed. Res. Int..

